# Advances in the molecular regulation mechanism of tumor dormancy and its therapeutic strategy

**DOI:** 10.1007/s12672-024-01049-2

**Published:** 2024-05-25

**Authors:** Yuan Wang, Linlin Wang, Yaojun Wei, Chuang Wei, Haohang Yang, Qiurui Chen, Rongxin Zhang, Han Shen

**Affiliations:** School of Life Sciences and Biopharmaceutics, Guangdong Pharmaceutical University, Guangzhou, 51006 People’s Republic of China

**Keywords:** Malignant tumors, Tumor metastasis, Tumor dormancy, Cell cycle block, Therapeutic strategies

## Abstract

Tumor dormancy is a stage in the growth and development of malignant cells and is one of the biological characteristics of malignant cells. Complex transitions involving dormant tumor cells between quiescent and proliferative states pose challenges for tumor eradication. This paper explores the biological features and molecular mechanisms of tumor dormancy and highlights emerging therapies. The strategies discussed promise innovative clinical potential against malignant tumors. Understanding the mechanisms of dormancy can help provide valuable insights into the diagnosis and treatment of malignant tumors to advance the fight against this world problem.

## Introduction

In recent decades, the rising global incidence of malignant tumors has become a considerable public health challenge. Projections from the 2020 Global Cancer Statistics [1] anticipate a 47% increase, reaching 28.4 million newly diagnosed cases annually by 2040. As the leading cause of global mortality, cancer impedes improvements in life expectancy. Metastasis, a significant contributor to tumor-associated deaths, is a formidable challenge in oncology [2]. Recent research underscores the importance of tumor dormancy in the metastatic process, where cancer cells, remaining inactive for extended periods, contribute to recurrence [3]. These resistant cells survive in a dormant state and hide for years or decades, ultimately giving rise to incurable metastases [4]. Over nine decades since its inception, the concept of tumor dormancy continues to challenge efforts in addressing metastatic recurrence [5, 6]. Understanding its molecular regulatory mechanisms is crucial for crafting innovative therapeutic strategies and advancing precision medicine to avoid over-diagnosis and over-medication in cancer cases.

## What is tumor dormancy?

### Definition of tumor dormancy

In 1934, Rupert Willis, as documented by his research [7], discerned from the data on late metastases in tumor patients that neoplastic cells undergo dormancy within tissues, leading to the formulation of the concept of dormant tumor cells, also known as Dormant Cancer Cells. By 1954, Geoffrey Hadfield [8] postulated that the recurrence of tumors is attributed to dormant cancer cells entering a phase of “temporary mitotic arrest,” characterizing these cells as non-proliferative entities undergoing G0/G1 phase cell cycle arrest.

**Fig. 1:**
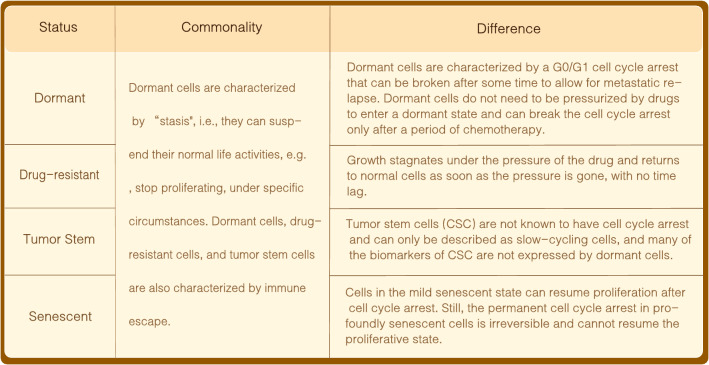
Similarities and differences between different types of dormant states

The interval during which an organism undergoes a state of reduced metabolic activity, known as stasis, is a mechanism employed to endure adverse conditions and enhance survival prospects [9]. Dormant cancer cells, characterized by cell cycle arrest, align with a stasis-like profile [9–11]. This profile encompasses drug-resistant persistent cells and tumor stem cells(Fig. 1), wherein the former halt under drug-induced stress, resuming normalcy upon stress cessation [12]. Dormant cancer cells may enter dormancy pre-treatment, deferring metastasis or recurrence post-treatment [13]. While quiescence and immune evasion are emerging traits of cancer stem cells (CSC) [14], evidence of direct cell cycle arrest in CSC remains inconclusive, denoting them as slow-cycling entities. Furthermore, molecular biomarkers expressed in CSC may not universally correlate with those in dormant cells [13], emphasizing that not all cells manifesting “stagnant” characteristics qualify as dormant cancer cells.

### Biological characteristics of tumor dormancy

**Fig. 2:**
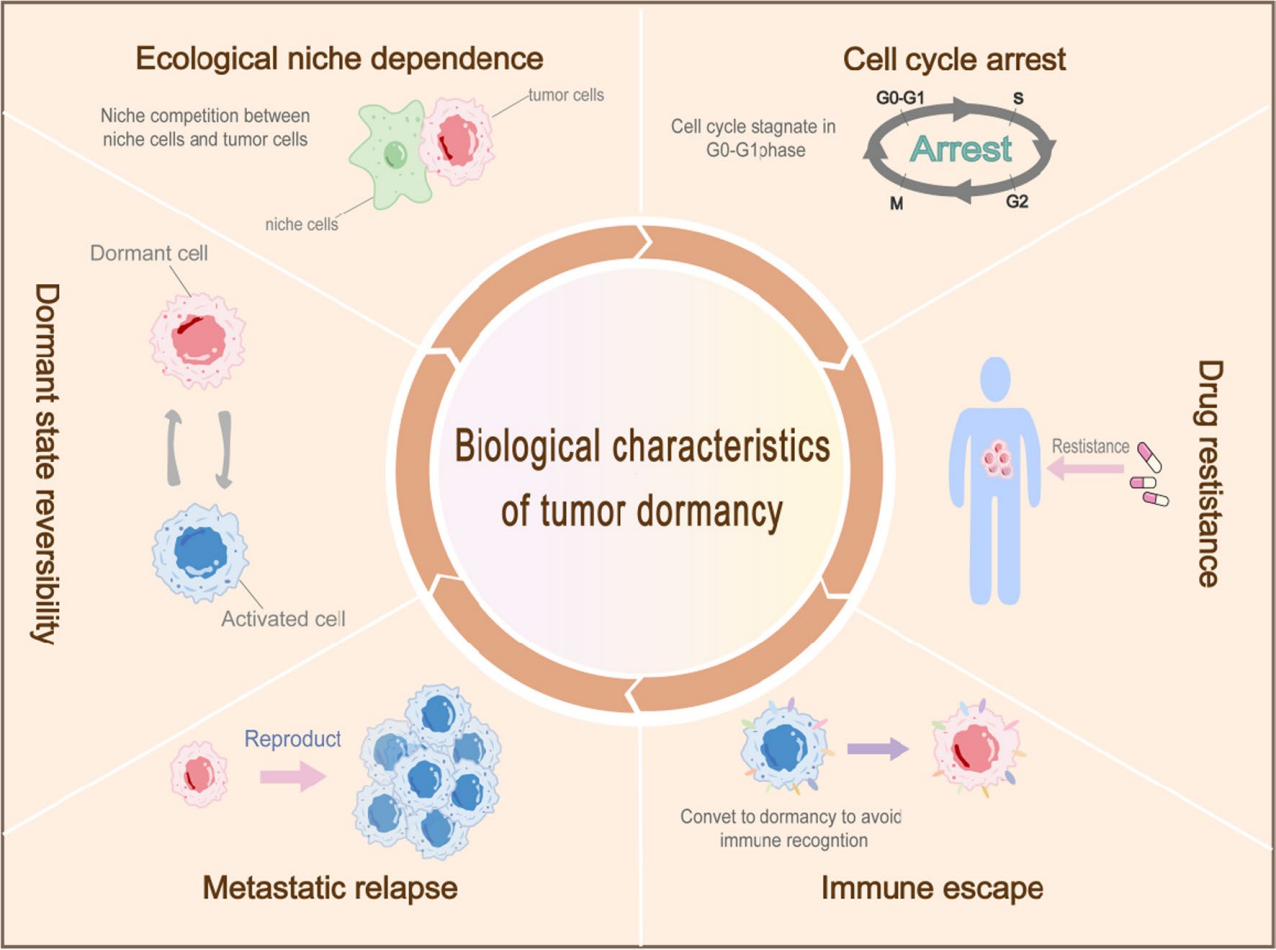
Main biological characteristics of tumor dormancy

Currently, the biological aspects of tumor dormancy can be attributed to the following six points [13](Fig. 2): 1. ecological site dependence; 2. cell cycle arrest; 3. drug resistance; 4. immune escape; 5. metastatic recurrence; and 6. reversibility of the dormant state.

#### Ecological niche dependence

In recent research, the proposition has emerged that the establishment of a primary tumor-modified pre-metastatic niche (PMN) fosters a microenvironment conducive to the survival and proliferation of dormant tumor cells [15]. The development of the PMN is contingent upon a sequential cascade of events, encompassing clot formation, vascular disruption, extracellular matrix alterations, cellular reprogramming of resident and recruited immune cells, and the up-regulation of pro-inflammatory molecules (e.g., S100, TNF-α, and TGF-β). Substantiated by a mounting body of clinical evidence, the existence of PMN has been observed in diverse sites among cancer patients, including lymph nodes and distant tissues such as the omentum and liver [16].

A study conducted by McGrath [17] et al. demonstrated that the transition of breast cancer cells between the dormant and activated states is contingent upon the specific characteristics of the ecological niche in which they colonize. Specifically, the colonization of an environmental cavity abundant in osteoblasts, accompanied by low oxygen levels and elevated calcium levels, was found to induce a dormant state in the tumor cells. In contrast, colonization of a perivascular ecological niche enriched with hematopoietic stem cells promoted active proliferation and facilitated the formation of metastatic lesions. In a study conducted by Lawson MA [18] et al., the behavior of individual myeloma cells was meticulously tracked through live imaging, leading to the validation of their colonization within the endosteal niche. Furthermore, it was observed that these myeloma cells entered a state of dormancy upon colonization, followed by subsequent activation that facilitated the formation of cellular colonies. Khoo WH et al. [19] observed that dormant myeloma cells persist within a distinct ecological niche in the bone microenvironment for prolonged periods, posing significant challenges to treatment strategies by evading immune surveillance and developing resistance to cytotoxic chemotherapy. Reactivation of these dormant cells is implicated in disease recurrence. The reliance of dormant tumor cells on ecological niches is a critical feature closely associated with their transition between dormant and activated states before metastasis. However, the molecular regulatory mechanisms of ecological niches in tumor dormancy remain unclear.

#### Cell cycle arrest

The passage elaborates on recent findings regarding the diverse dormancy states exhibited by disseminated tumor cells (DTCs), particularly those originating from breast tumors. These states encompass cellular dormancy, characterized by solitary, quiescent cells, and tumor mass dormancy, typified by small cell clusters that maintain tumoral homeostasis through a delicate balance between proliferation and death, often termed dormant micrometastasis [20]. These DTCs are predominantly identified as tumor cells undergoing G0/G1 phase cell cycle arrest, representing a subpopulation in a state of relative quiescence. However, such cell cycle arrest is not exclusive to tumor dormant cells; drug-resistant persistent cells evade cytotoxic stress by undergoing cell cycle arrest or entering a slow-cycling state post-drug stimulation [21].

The nonproliferative or slow-cycling phase exhibited by disseminated tumor cells (DTCs) implies an inevitable period of G0/G1 blockade during this biological state. However, the collection of dormant signature genes associated with late breast cancer recurrence comprises several genes that remain differentially regulated in cells lacking growth factors [22]. These differentially regulated genes encompass those involved in the regulation of quiescence in normal stem cells, exemplified by approximately 62% of genes responsible for inducing dormancy in normal muscle, hair follicle, and hematopoietic stem cells being upregulated in dormant head and neck squamous cell carcinoma cells [23]. Multiple factors contribute to the occurrence of cell cycle arrests, including signaling pathways induced by cytokines and the presence of a hypoxic microenvironment, both of which can impede tumor cell proliferation and induce cell cycle arrest within the G0/G1 phase [24].

#### Drug resistance

Dormancy represents a fundamental survival strategy employed by organisms to endure adverse conditions, thereby conferring a selective advantage [25]. In the context of tumors, the utilization of dormancy serves as a conservative mechanism to enhance resistance against anticancer drugs, allowing tumor cells to withstand the onslaught of chemotherapy and subsequent clearance attempts, thereby ensuring their survival.

The recurrence and metastasis of tumor cells post-treatment may be attributed to the ability of dormant tumor cells to evade drug effects, thus surviving drug attacks and leading to recurrence. Most anticancer therapies target actively dividing cells, making dormant cells less susceptible to the selective pressure exerted by these drugs, as they inhibit mechanisms associated with proliferation, which is characteristic of dormancy [26]. Further studies have validated these findings across various tumor types, including breast cancer, glioblastoma, melanoma, and prostate cancer. For instance, glioblastoma cells exposed to the DNA methylating agent temozolomide demonstrated the emergence of a distinct subpopulation of dormant cells with regenerative potential, ultimately contributing to tumor recurrence [27]. Moreover, research by Patton EE et al. [28] has shown that a considerable portion of melanoma patients display inherent resistance to existing therapeutic approaches, with a notable subset of initially responsive patients eventually developing acquired resistance upon relapse. The development of drug resistance in tumor dormant cells primarily occurs through primary and acquired mechanisms, necessitating tailored therapeutic strategies to address the diverse pathways of tumor cell resistance.

#### Immune escape

Payne KK [29] et al. conducted experiments employing adriamycin (ADR) treatment on MMC tumor cells to induce a state of isolated tumor dormancy. Subsequently, dormant tumor cells were subjected to interferon-gamma (IFN-γ), an anti-tumor T-cell response product, to evaluate the responsiveness of distinct types of dormant cells (resting and inert) towards immune editing. The results confirmed that inert tumor dormant cells elicited immune editing processes leading to immune escape while resting dormant cells did not demonstrate a similar immune response.

The cancer immune response can be characterized by a sequential progression involving three distinct phases, as elucidated in the Immunology of Disease editorial [30]. The initial phase, termed the elimination phase, represents a period during which the immune system actively identifies and eliminates malignant cells through effective recognition and targeted cytotoxicity. Following this phase, a transitional period known as the equilibrium phase ensues, wherein tumor cells persist within the organism but remain dormant, largely constrained by immune-mediated restrictions. Subsequently, during the escape phase, dormant tumor cells adapt to a specialized ecological niche, evading immune surveillance and reactivating their proliferative capacity, thereby bypassing immune control mechanisms. This immune evasion ability facilitates tumor metastasis and recurrence. Notably, inert tumor dormant cells possess immune escape characteristics, underscoring the importance of identifying and targeting these cells to reduce the likelihood of tumor recurrence during treatment.

#### Metastatic relapse

Metastasis, the dissemination of tumor cells from the primary site to distant locations in the body, represents a critical factor contributing to the majority of cancer-related fatalities [27]. The intricate interplay between dormant cancer cells and the inflammatory milieu plays a pivotal role in the reactivation and progression of latent tumor cells toward cryptic metastatic lesions, consequently underpinning the phenomenon of tumor recurrence. In the context of persistent inflammation, neutrophils present within the lung microenvironment demonstrate the capacity to generate extracellular traps composed of a combination of DNA and cytotoxic proteins and proteases. These extracellular traps possess the capability to activate quiescent cancer cells through the remodeling of the adjacent extracellular matrix, thereby instigating the formation of micrometastases [31].

Metastasis represents a multifaceted and sequential process encompassing several intricate steps whereby DTCs traverse diverse cellular states, including migration, quiescence, and proliferation. This intricate journey entails the transition of DTCs across multiple microenvironments, such as the tumor extracellular matrix, blood vessels, and lymphatic vessels, ultimately culminating in the establishment of secondary lesions within distant organs such as the lung, bone, brain, and liver [32]. Solid tumors exhibit a predilection for metastatic colonization in distinct anatomical sites, primarily involving visceral organs and the skeletal system. Skeletal metastases commonly manifest in patients afflicted with multiple myeloma (up to 3% incidence) and breast cancer (65–95% incidence) and particularly demonstrate higher proclivity in the case of triple-negative breast cancer (TNBC). Conversely, internal organs are more prone to metastatic involvement in TNBC cases [13]. Ban [33] et al. elucidated the multistep nature of metastatic recurrence, encompassing a minimum of four distinct stages. Initially, tumor cells navigate towards a conducive ecological niche where they can establish colonization. Subsequently, these tumor cells undergo adaptive changes to acclimate to the local microenvironment, entering a dormant state characterized by quiescence. Following this period of dormancy, the dormant tumor cells undergo reactivation, initiating proliferation and growth. Ultimately, the activated tumor cells orchestrate the disruption of the original ecological niche's structural and functional integrity, further perpetuating their metastatic progression.

#### Dormant state reversibility

"Tumor dormancy" describes a coordinated mechanism where cancer cells undergo reversible cell cycle arrest, typically at the G0/G1 phase, driven by activation of a quiescent program. Dormant cells exhibit specific features, such as the ability to halt division while retaining the capacity to re-enter the cell cycle later. This reversibility is a defining trait of tumor dormancy, underscoring its dynamic nature and the potential for dormant cells to re-enter active proliferation [25]. Drug-resistant persistent cells share some characteristics with dormant tumor cells in terms of reversibility; however, they differ in that removal from the drug environment promptly ends their dormancy, restoring sensitivity to the drug [34].

The regulation of tumor cell dormancy reversibility encompasses intracellular and external environmental factors. Lawson et al. [18] demonstrated the dynamic nature of dormancy, showing its induction via interactions with bone lining cells or osteoblasts, and cessation through osteoclast-mediated endosomal ecotone remodeling. Importantly, this process is reversible, indicating dormant cells' capability to transition between dormant and active states in response to the bone microenvironment's dynamic interplay. Han [13] et al. postulated two potential mechanisms for disrupting the dormant state of tumor cells. Firstly, dormant tumor cells may accumulate an adequate nutrient reservoir, facilitating their progression toward proliferation. Secondly, alterations in the external milieu can trigger dormant tumor cells to undergo activation and escape from the dormant state. These proposed mechanisms shed light on the dynamic interplay between internal cellular processes and external environmental cues in regulating the transition from dormancy to active proliferation in tumor cells.

### Biomarkers of tumor dormancy

Presently, Wu [35] et al. have documented the expression of NR2F1, a recognized marker of tumor dormancy, in breast cancer-associated fibroblasts. They observed that heightened NR2F1 expression in these fibroblasts impedes cell proliferation and metastasis-related pathways. Additionally, Yumoto [36] et al. identified elevated AXL levels, a tyrosine kinase receptor for growth arrest-specific 6 (Gas6), in dormant disseminated tumor cells (DTC). Their findings revealed a positive correlation between AXL expression, TGF-β, and its receptor in disseminated prostate cancer cells, indicating a crucial crosstalk between TGF-β and the Gas6/Axl signaling pathways that regulate tumor dormancy.

Moreover, a prominent characteristic of tumor dormancy is the arrest of the cell cycle in the G0/G1 phase. Consequently, markers associated with cell cycle arrest emerge as potential indicators of tumor dormancy. Notably, p27 and p21 have been implicated in troglitazone-induced cell cycle block in human hepatocellular carcinoma cells [37]. Studies by Sosa MS and colleagues [38] revealed that the ERKlow/p38high phenotype induces cell cycle arrest in the G0/G1 phase, suggesting that p38 activation can prompt tumor cells to enter or sustain a dormant state.

In their research, Di Martino JS and colleagues [39] employed the dormancy marker p27 to induce a dormant state in tumor cells. Similarly, Fane ME and colleagues [40], in a study published in Nature, utilized NR2F1, Axl, Gas6, p27, p21, and p38 as dormancy markers. Their investigation confirmed that WNT5A contributes to the maintenance of cellular dormancy, promoting survival and adaptation within the lung microenvironment. The study concluded that WNT5A serves as a significant marker of tumor dormancy. While many of the tumor dormancy markers mentioned above have been linked to tumor dormancy mechanisms, there is currently no method for accurately detecting dormant cells in patients using biomarkers in clinical trials.

## Molecular regulatory mechanisms of tumor dormancy

**Fig. 3:**
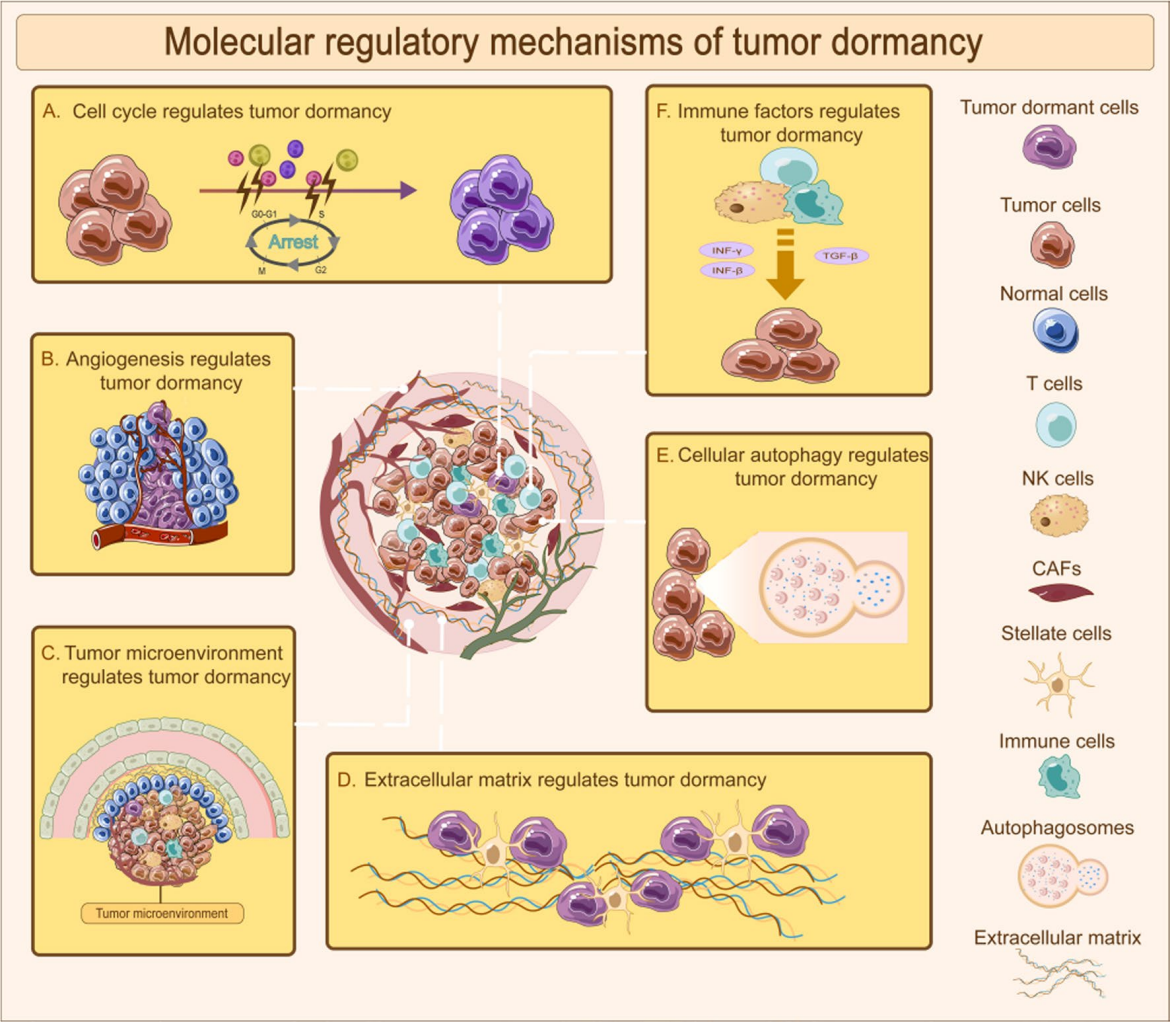
Main molecular regulatory mechanisms of tumor dormancy

The establishment and sustenance of tumor dormancy entail intricate molecular regulatory mechanisms that encompass various factors, including the cell cycle, tumor angiogenesis, tumor microenvironment, extracellular matrix, cellular autophagy, and immune elements(Fig. 3). These mechanisms operate in conjunction, orchestrating the dynamic equilibrium of tumor dormancy. Notably, the intricate interplay of these factors is intimately linked to external stimuli and the specific characteristics inherent to tumor cells, contributing to the complex and context-dependent nature of tumor dormancy.

### Cell cycle

**Fig. 4:**
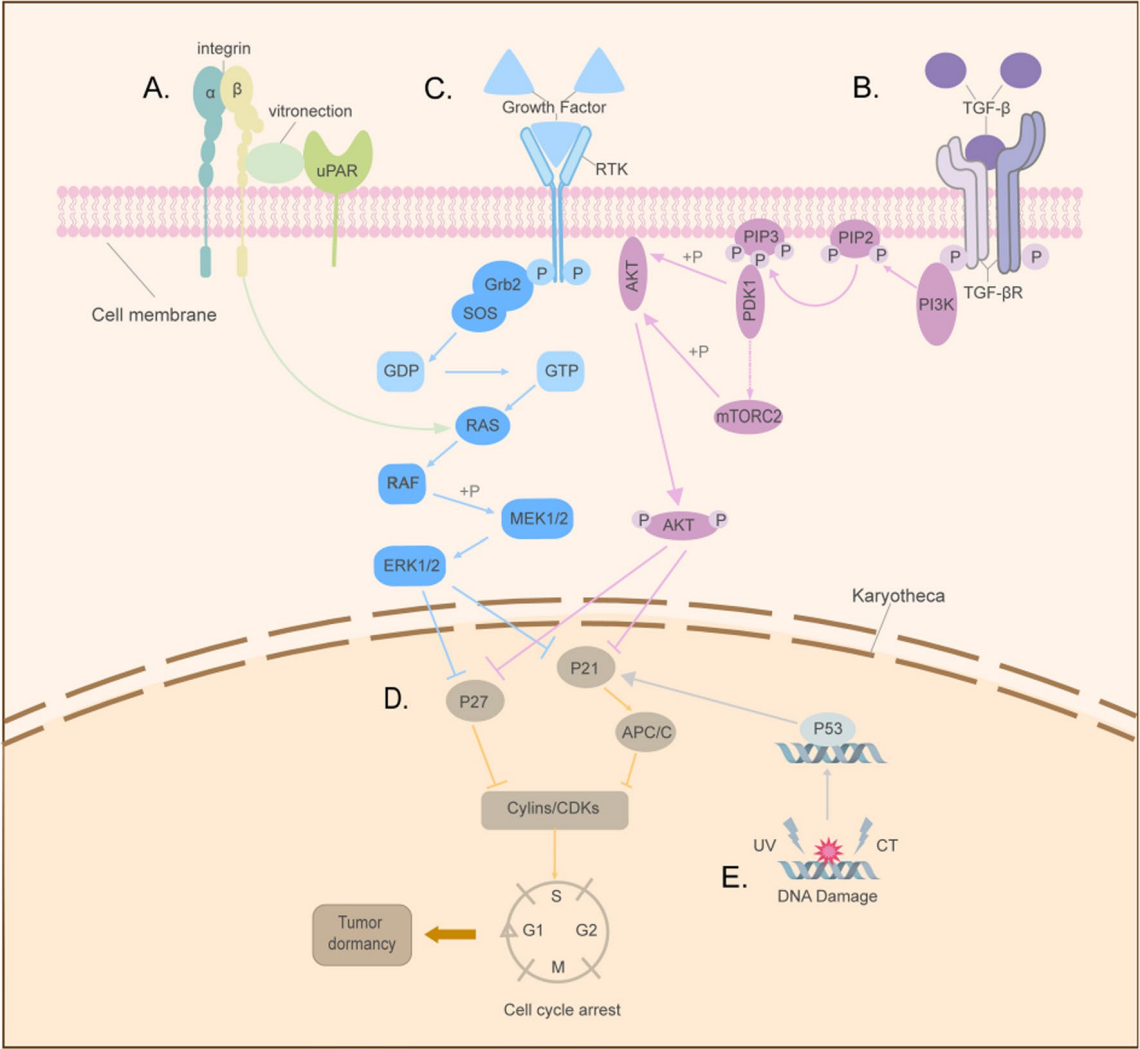
Illustrates the regulatory mechanisms of tumor dormancy on the cell cycle. **A** The uPAR-integrin complex activates Ras, which in turn activates Raf through phosphorylation. Activated Raf phosphorylates MEK1/2, leading to the activation of ERK1/2. ERK1/2 translocates to the nucleus and inhibits the expression of P21/P27, thereby disrupting the formation of the Cyclin-CDK complex and inducing cell cycle arrest. **B** TGF-β binding to TGF-β leads to tyrosine phosphorylation, recruiting PI3K. Activated PI3K converts PIP2 to PIP3, recruiting 3-phosphoinositide-dependent protein kinase 1(PDK1) and Akt. Akt activation regulates the mammalian target of rapamycin complex 2 (mTORC2) activity, leading to full Akt phosphorylation and its translocation to the cytoplasm. **C** Ligand binding to the receptor tyrosine kinase(RTK) on the cell membrane triggers receptor dimerization and activation of its tyrosine kinase domain. This activates Grb2, which in turn activates SOS, resulting in the activation of the Ras-Raf-MEK-ERK/MAPK pathway and subsequent cell cycle arrest. **D** CDKs mediate the G0/G1 to S phase transition, while P21/P27 inhibit CDK activity. Elevated P21/P27 expression disrupts Cyclin-CDK complex formation, leading to cell cycle arrest. **E** DNA damage induced by chemotherapy upregulates p53 expression, which binds to the p21 promoter and activates p21 gene transcription, resulting in the activation of the p21-Cyclin-CDK signaling pathway and cell cycle arrest

Cell cycle arrest plays a pivotal role in tumor dormancy, often involving alterations in cell cycle-related pathways. The interaction between urokinase plasminogen activator receptor (uPAR) and α5β1 integrin, facilitated by bilirubin, leads to the formation of the uPAR-α5β1 complex. Subsequently, this complex activates the Ras-ERK (Extracellular signal-regulated kinase) pathway, promoting the in vivo proliferation of tumor cells [41].Consequently, by blocking the interaction of this complex, the activation of the Ras-ERK signaling pathway is attenuated, resulting in the suppression of tumor cell proliferation (Fig. 4A). Various growth factors, such as TGF-β, engage with tyrosine kinase receptors, initiating receptor dimerization and subsequent tyrosine phosphorylation events. Upon tyrosine phosphorylation, the phosphoinositide 3-kinase—protein kinase B (PI3K-Akt) signaling pathway [42] is activated. Phosphorylated tyrosine recruits PI3K, which catalyzes the conversion of phosphatidylinositol 4,5-bisphosphate (PIP2) to phosphatidylinositol 3,4,5-trisphosphate (PIP3) on the cell membrane. This leads to subsequent Akt phosphorylation, facilitating downstream signaling events involved in cell survival, growth, and metabolism. Qiu [42] et al. demonstrated that following PI3K-Akt phosphorylation, the activation of transcription factors downstream of the PI3K/Akt signaling pathway, known to be associated with tumor cell proliferation, is enhanced, thereby promoting tumor cell proliferation. Moreover, phosphorylated tyrosine triggers the activation of the MEK-ERK/MAPK [43] signaling pathway (Fig. 4B). The phosphorylated tyrosine residue on the receptor engages with the SH2 domain of Grb 2(growth factor receptor binding protein 2) present on the cytosolic membrane. Simultaneously, the SH3 domain of Grb2 interacts with the guanylate exchange factor SOS (Son of Sevenless), thereby initiating the activation of the MEK-ERK/MAPK signaling pathway(Fig. 4C).

The uPAR-α5β1-Ras-ERK [41], PI3K-Akt [42], and MEK-ERK/MAPK [43] pathways collectively modulate the p27/p21-Cyclin- cyclin-dependent kinases (CDKs) [26] signaling pathway (Fig. 4D) to induce cell cycle arrest. These signaling pathways intricately modulate the expression and activity of critical regulators involved in cell cycle control, including p27, p21, cyclins, and CDKs, ultimately leading to the arrest of cell cycle progression. Notably, the diminished phosphorylation of ERK or Akt, particularly in the low-expressed state, induces the upregulation of P21 and P27 expression. Consequently, this inhibits the formation of the cyclin-dependent kinase-cyclin complex, impeding the transition from the G0/G1 phase to the S phase and resulting in cell cycle arrest, thus facilitating the entry of DTCs into a dormant state [26].

Upon exposure to chemotherapeutic agents, DNA damage is elicited, triggering the activation of the p53-p21-APC/C-dependent pathway [44] (Fig. 4E). Notably, the administration of pentafluorouracil (5-Fu) to tumor cells induces DNA damage, prompting p53 accumulation within the nucleoplasm and facilitating specific DNA binding. Functioning as a transcription factor, p53 binds to the p21 promoter region, thereby initiating p21 gene transcription. Consequently, p21 expression is upregulated, leading to the activation of the p21-Cyclin-CDKs [26] signaling pathway (Fig. 4D), ultimately culminating in cell cycle arrest.

### Senescence

The aforementioned pathways directly influence the cell cycle, leading tumor cells to arrest and enter a quiescent state. Notably, the relationship between tumor senescence, a reversible phenomenon, and tumor dormancy is contentious. Different triggers of tumor senescence have varied effects on the formation of tumor dormancy. We propose that mechanisms enabling escape from senescence may be crucial in both maintaining and breaking dormancy.

Senescence can be driven by multiple stressors, including replication restriction (replicative senescence, RS) [45], oncogene activation (oncogene-induced senescence, OIS) [46], oxidative stress [47], and therapeutic interventions (treatment-induced senescence, TIS) [48]. Notably, DNA damage is not always necessary for senescence induction. Instances of senescence induction without DNA damage have been documented, including cases of TIS induced by targeted drugs such as CDK4/6 inhibitors [49], androgen receptor antagonists [50], and aurora kinase inhibitors [51].

Currently, the mechanisms behind escaping oncogene-induced senescence (OIS) remain unclear. Recent investigations in colorectal cancer have unveiled the pivotal roles of AP1 and POU2F2 in OIS evasion, identifying the epigenetic memory of senescence-associated chromatin scarring (SACS) during colorectal cancer progression [52]. In contrast, authentic senescence signifies a permanent withdrawal from the cell cycle, challenging reactivation for recurrent tumor formation [53]. While reversible senescence is documented [54], screens involving RNA interference or CRISPR-Cas9 reveal genes facilitating senescence escape, with limited evidence supporting the actual escape from senescence [51]. Pro-senescence stress in the nucleus transmits signals to p21CIP1 via DNA damage response (DDR), activating the mitogen-activated protein kinase cascade and upregulating p16^INK4a^ expression. Both p16^INK4a^ and p21CIP1 induce G1 phase cell cycle arrest, and cells with low p16^INK4a^ levels may resume proliferation following acute p53 inactivation [55, 56]. These findings suggest the existence of a state of mild senescence characterized by low p16INK4a levels, in contrast to a profound senescent state.

“Quiescence” is often correlated with dormancy, as dormant cells are typically viewed as being in a state of quiescence [57]. Quiescent cells are in a state of growth arrest in the G0 phase and resume proliferation shortly after the removal of the growth arrest-inducing stimulus. In contrast to quiescent cells, senescent cells are believed to undergo repression in transcription and metabolism [58]. While quiescence may represent a "dormant" growth arrest mechanism for tumor cells, senescence may represent another process. Senescent cells enter a prolonged period of growth arrest, similar to the concept that dormant tumor cells do not increase or cycle slowly. Moreover, dormant tumor cells, particularly those at undeveloped metastatic sites, are likely to have acquired the ability to survive environmental stresses that would otherwise result in cell death. This is supported by the fact that an increased cellular resistance to apoptosis is a hallmark of the senescence program [59]. Senescence is characterized by elevated expression of anti-apoptotic genes Bcl2 and Bcl2l1, leading to reduced sensitivity to various apoptosis-inducing stimuli such as chemotherapy [60]. However, it is challenging to consider senescence as another form of dormant growth arrest, as dormant cells are believed to have the ability to increase and may contribute to the reemergence of fully grown tumors [61]. Traditionally, senescence was viewed as a permanent cellular state, contrasting with prior assumptions about dormancy. However, recent evidence suggests that senescent tumor cells are not restricted to indefinite arrest and that senescence-related growth arrest can be resolved [62].

Over the past two decades, accumulating evidence has demonstrated the escape from therapy-induced senescence (TIS). Key experiments by Daouillon et al. [63] have utilized a subpopulation of chemotherapy-induced senescent cells to show that these cells can escape growth arrest and resume proliferation. Specifically, using the irinotecan-induced colon senescence model, they observed reductions in senescence markers, such as SA-β-gal, p21Cip1, and γH2AX, as well as promyelocytic leukemia nuclei (PML) vesicles. The cell population consisted of 70% senescent and 30% proliferating cells, indicating successful escape from senescent growth arrest. Milanovic et al. [64] demonstrated senescence escape in a lymphoma model exposed to adriamycin, showing that loss of p53 or Suv39h1 function promotes senescence escape in lymphoma cells. Additionally, Duy et al. [65] observed senescence escape in progenitor cells, with surviving senescent subpopulations able to repopulate after two months. These findings provide a solid experimental basis for understanding TIS escape and the mechanisms underlying tumor cell escape from senescence growth arrest.

### Angiogenesis

Tumor progression is commonly associated with angiogenesis, a process involving the formation of new blood vessels, which facilitates the infiltration of malignant cells into the circulatory system, supporting their survival and growth [66]. Angiogenesis, the process of blood vessel formation, is regulated by a balance between pro-angiogenic and anti-angiogenic factors. Prior to the angiogenic switch, anti-angiogenic factors prevail, resulting in limited oxygen and nutrient supply to distant areas from pre-existing blood vessels, leading to cell death. Following the angiogenic switch, pro-angiogenic factors become dominant, facilitating tumor proliferation. The state of angiogenic dormancy arises from an equilibrium between cancer cell proliferation and death [1], leading to tumor mass dormancy controlled by the balance between proliferation and apoptosis rates in micrometastatic lesions [39]. This dormancy type, distinct from DTCs cell dormancy, is maintained by anti-angiogenic signaling regulation [67]. Tumor progression, recurrence, and metastasis depend on new blood vessel formation to supply nutrients, growth factors, and oxygen. Therefore, inhibiting tumor-associated angiogenesis can induce tumor dormancy [68].

This characteristic can be clinically advantageous, as the use of angiogenesis inhibitors can induce tumor dormancy, potentially lowering the risk of tumor reactivation years after diagnosis. Extracellular metalloproteinase-inducible factor (EMMPRIN), also known as CD147, has been identified as a crucial facilitator of angiogenesis. Feigelman et al. [69] proposed its role in preventing tumor cells from entering dormancy. Their study involved knocking down EMMPRIN expression in CT26 cells, which led to a decreased pERK/pP38 ratio, suggesting a potential mechanism for inducing dormancy.This downregulation of EMMPRIN led to the upregulation of waveform proteins, EMT-inducible transcription factors, and dormancy markers, ultimately suppressing the proliferation and angiogenic potential of the cells and inducing them into a dormant state. These findings suggest that EMMPRIN plays a critical role in modulating cellular dormancy and angiogenesis, highlighting its potential therapeutic target for inhibiting tumor progression and promoting dormancy in cancer cells.

### Tumor microenvironment

Tumor masses consist of cancer cells along with a diverse array of host cells, secretory factors, and extracellular matrix components, collectively referred to as the tumor microenvironment (TME). The progression of tumors is heavily influenced by the intricate interactions between cancer cells and their surrounding environment. These interactions play a pivotal role in determining the fate of the primary tumor, whether it undergoes eradication, metastasis, or establishes dormant micrometastases. The dynamic interplay within the TME governs the behavior and outcomes of cancer cells, highlighting the significance of understanding and targeting the tumor microenvironment for effective therapeutic interventions [39].

The interplay between the tumor microenvironment and intrinsic factors of tumor cells significantly influences the transition from tumor dormancy to activation. Studies indicate that the tumor microenvironment plays a pivotal role in regulating tumor dormancy, particularly through the integrin signaling pathway. Notably, Integrin β1 emerges as a critical regulator of tumor cell dormancy, exerting its influence within the tumor microenvironment. Mechanistically, Integrin β1 initiates downstream signaling pathways involving phosphorylated Src (p-Src) and phosphorylated focal adhesion kinase (p-FAK), subsequently activating phosphorylated extracellular signal-regulated kinase (p-ERK) and phosphorylated p38 (p-p38).These activated signaling molecules further stimulate myosin light chain kinase (MLCK), resulting in cytoskeletal reorganization and the promotion of metastatic growth [70]. This process enables tumor cells to escape dormancy, leading to metastasis and tumor progression. Conversely, downregulating the expression of integrin β1 and its downstream signals can induce tumor cells to maintain a dormant state, offering potential therapeutic strategies to regulate tumor dormancy by targeting the integrin signaling pathway within the tumor microenvironment.

### Extracellular matrix

The extracellular matrix (ECM) is a meshwork of proteins that anchor tumor cells and provide signals that regulate tumor cell behavior during metastasis and many ECM-bound factors involved in solid tumors' dormancy induction [39, 71].

Albrengues [72] et al. conducted in vitro experiments and identified laminin−111, −211, −411, and −511 as crucial extracellular matrix (ECM) proteins involved in the activation of cancer cells by neutrophil extracellular traps (NETs), leading to the awakening of dormant cells. Their findings revealed that the matrix metalloproteinase MMP111, secreted by neutrophils, remodels the ECM protein laminin-9, thereby activating integrin α3β1 signaling. This activation disrupts dormancy and facilitates metastatic growth in cancer cells. These findings shed light on the mechanisms underlying ECM-mediated regulation of tumor dormancy and metastasis.

Dai [73] et al. demonstrated that individual DTCs occupy specific vascular niches, with quiescent DTCs localized at the astrocyte end feet. At these sites, astrocytes deposit laminin-211, which promotes DTC quiescence. This quiescent state is achieved by inducing the interaction between myostatin receptors and yes-associated protein (YAP), resulting in the sequestration of YAP away from the nucleus and the subsequent suppression of its pro-metastatic functions. YAP serves as a pivotal regulator of cell proliferation and apoptosis, contributing significantly to tumor senescence. It directly governs deoxynucleotide metabolism, crucial for cell growth and resistance to chemotherapeutic agents [74]. The absence of YAP impedes cell proliferation and triggers premature senescence, a process dependent on TEAD and Rb/p16/p53 pathways [75]. Additionally, nuclear accumulation of YAP enhances the survival of senescent cells by upregulating the anti-apoptotic gene surviving [76]. YAP’s involvement in accelerating vascular senescence is linked to inhibiting autophagic flux and activating the mTOR pathway [77]. These insights suggest that targeting YAP may hold promise in cancer therapy by inducing tumor senescence. These findings provide insights into the role of astrocyte-laminin interactions in regulating DTC quiescence and inhibiting metastatic progression.

Daniel [78] et al. demonstrated that modulation of ECM components could induce tumor cells into a dormant state through the inhibition of Wnt and Notch signaling pathways or the activation of the BMP signaling pathway. Additionally, the ratio of active ERK to p38 MAPK in the ECM has been identified as a key determinant of cellular dormancy [38]. The MAPK pathway, particularly the p38 MAPK and ERK1/2 signaling, plays a central role in regulating tumor dormancy across various cancer types, including breast, prostate, bladder, and melanoma. Dormant cells exhibit heightened p38 MAPK activity and decreased ERK1/2 activity, and the p38 MAPK high/ERKlow phenotype has emerged as a widely utilized marker for identifying the dormant state [79].

### Cellular autophagy

Autophagy, a cellular process involving lysosome-mediated degradation, is pivotal for protein breakdown, cytoplasmic organelle renewal, and recycling of cellular components [80]. Four functions of autophagy, cytoprotective, nonprotective, cytotoxic, and cytostatic, have been reported in the existing literature [81].

Autophagy typically serves a protective role, acting as a dynamic circulatory system to mitigate cellular damage and maintain in vivo homeostasis [82]. In cancer, autophagy plays a crucial role in sustaining cancer cells during adverse conditions such as starvation, hypoxia, fozen and chemotherapy/radiotherapy [83, 84]. This process offers protection against cell death and promotes cancer cell growth. However, the protective autophagy program of cancer cells can enhance their resistance to chemotherapeutic drugs, allowing some cells to survive drug pressure and lead to tumor repopulation, metastasis, or recurrence. Several studies have demonstrated that autophagy inhibitors can enhance the effectiveness of anticancer drugs. For example, Xi G et al. [85] found that paclitaxel-induced autophagy in A549 cells reduced the chemotherapy's efficacy, but this effect was reversed after pretreatment with the autophagy inhibitor 3-methyladenine (3-MA) or small interfering RNA targeting the autophagy gene beclin1, which enhanced cancer cell death induction. Additionally, Lin JF [86] and colleagues showed that combining cisplatin with autophagy inhibitors could overcome cisplatin resistance mediated by autophagy induction. Tumor cells can influence innate immunity through autophagy, either directly or indirectly.

In contrast to protective autophagy, nonprotective autophagy does not serve as a cytoprotective mechanism. However, tumor cells can exploit autophagy to evade immune recognition during stress responses [81]. Baginska et al. [87] demonstrated that hypoxic tumor cells utilize autophagy to resist natural killer (NK) cell-mediated immune responses. In vitro, hypoxia-induced autophagosomes can degrade NK cell-derived granzyme B, impairing NK cell cytotoxicity against breast cancer cells. Inhibition of autophagy restores granzyme B levels, enhancing NK cell-mediated tumor cell killing. Zhang et al. [88] showed that autophagy in glioblastoma cells inhibits phagocytosis by macrophages. Inhibition of autophagy increases macrophage infiltration and promotes tumor cell apoptosis.

Additionally, the clinical implications of autophagy’s cytotoxic and cytoprotective properties are noteworthy. Inhibition of cytoprotective autophagy sensitizes cells to therapeutic interventions, whereas inhibition of cytotoxic autophagy reduces sensitivity to treatments [81]. Yang et al. [89]demonstrated that ARCSP modulates the AMPK/mTOR autophagy pathway, blocking autophagosome-lysosome fusion and hindering endosomal maturation, thereby enhancing cytotoxicity. Bristol et al. [90] found that vitamin D combined with radiation induces cytotoxic autophagy in breast cancer, leading to tumor cell death. In contrast, cytostatic autophagy, which promotes cell survival by inducing growth arrest, did not result in cell death in their study [81]. Sharma et al. [91] showed that 1,25-D3, the active form of vitamin D, and its analog EB 1089, kill A549 and H460 non-small cell lung cancer (NSCLC) cells, prolonging radiation-induced growth arrest and inhibiting proliferative recovery. Zhang et al. [92] demonstrated that berberine inhibits human gastric cancer cell growth in vitro and in vivo by suppressing MAPK/mTOR/p70S6K and Akt-mediated autophagy.

Notably, dormant tumor cells, exemplified in various cancer types, exhibit heightened autophagic activity, as validated through in vitro, ex vivo, and xenograft models [93, 94]. La Belle Flynn et al. [94] investigated the differential expression of the glycolytic gene Pfkfb3 in metastatic breast cancer cells compared to dormant cells. Their findings revealed that Pfkfb3 is expressed in metastatic breast cancer cells but absent in dormant cells. Importantly, they experimentally established a negative correlation between PFKFB3 expression and autophagy. Dormant breast cancer cells exhibited a PFKFB3_low_/autophagy^high^ profile, transitioning to PFKFB3^high^ /autophagy_low_ upon breast cancer recurrence. This underscores the significant association between autophagy, tumor dormancy, and metastatic recurrence. Notably, the researchers demonstrated that inactivating autophagy through the targeting of Atg3, Atg7, or p62/sequestosome-1 led to enhanced PFKFB3 expression, subsequently restoring breast cancer cell proliferation.Sutton MN et al. [95] conducted experiments involving amino acid deprivation on human ovarian cancer cells, revealing a consequential inhibition of mTOR expression. This inhibition prompted the dissociation of E2F1/E2F4 from the DIRAS3 promoter, leading to an up-regulation of DIRAS3 expression. Consequently, DIRAS3-mediated autophagy was enhanced, enabling the maintenance of human ovarian cancer cell growth in a state of growth homeostasis under stress. Treatment with chloroquine, a functional autophagy inhibitor, significantly reduced the growth of dormant ovarian cancer cells post down-regulation of DIRAS3 expression, emphasizing the crucial role of autophagy in the persistence of dormant cancer cells post-initial surgery and chemotherapy [93].

### Immune factors

The interplay between immune signaling and tumor cells exerts regulatory control over tumor dormancy. Extensive evidence supports the role of IFN-γ and IFN-β in modulating tumor dormancy across diverse cancer types, including breast cancer [96], bladder cancer [42], and melanoma [97]. IFN-γ and IFN-β mediate tumor dormancy via the IDO-Kyn-Ahr-P27 pathway [42, 97]. Upon exposure to IFN-γ and IFN-β, tumor-regenerating cells exhibit elevated expression of indoleamine 2,3-dioxygenase (IDO) and aromatic hydrocarbon receptor (AHR). Subsequently, the upregulation of IDO leads to the production of abundant endogenous kynurenine (Kyn), which acts as the endogenous ligand for AHR. The binding of Kyn to AHR activates the nuclear translocation of AHR, which in turn modulates the binding of the cell cycle regulator protein P27. This regulatory event triggers cell cycle arrest through the p27-Cyclin-CDKs signaling pathway [26], effectively inducing a dormant state in tumor-regenerating cells.

Lan Q [98] et al. demonstrated that high doses of adriamycin and methotrexate chemotherapeutic agents induced immunodormancy in estrogen receptor-negative (ER) breast cancers through the IFN-β/IFNAR/IRF7 pathway. Residual breast cancer cells responded to the treatment by entering a quiescent state characterized by the expression of IFN-β. Conversely, cancer cells that escaped dormancy following chemotherapy displayed diminished secretion of IFN-β. These findings provide direct evidence supporting the crucial role of IFN-β expression in maintaining immunodormancy in breast cancer cells(ER), highlighting its potential as a therapeutic target for regulating tumor quiescence.

TGF-β signaling constitutes a pivotal component in the regulation of tumor dormancy. Existing evidence supports the notion that TGF-β governs the dormant characteristics of both normal stem cells and disseminated tumor cells through the molecular pathway involving TGF-β2 signaling activation of MAPK p38α/β. This activation leads to the induction of low-signaling ratios of ERK/p38 and down-regulation of CDK4, consequently promoting the dormancy of malignant tumor cells [99].

## Tumor treatment strategies based on tumor dormancy characteristics

**Fig. 5:**
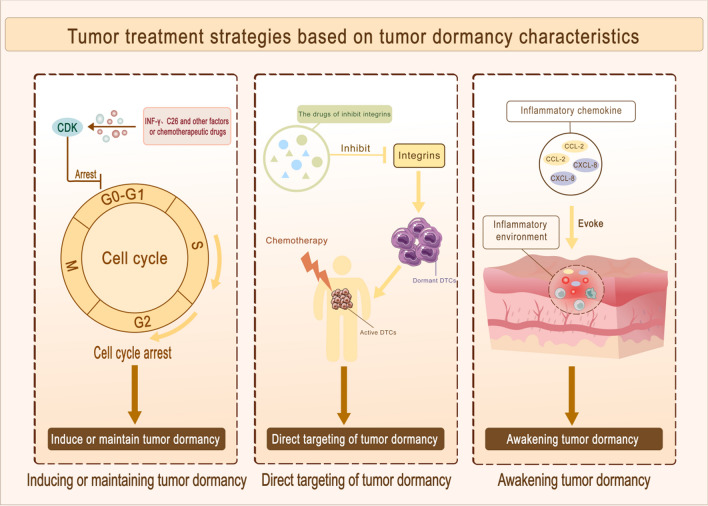
Tumor treatment strategy based on tumor dormancy

Tumor dormancy can have positive and negative effects on patient prognosis, including the development of drug resistance, recurrence, and metastasis, as well as slowed tumor progression due to cell cycle arrest. Developing therapeutic strategies based on tumor dormancy characteristics requires careful consideration of its influencing factors and patient adaptability. Current research is focused on several key areas, including the induction or maintenance of tumor cell dormancy to delay recurrence and create a chronic, controllable disease state; the direct targeting of dormant cells to activate the immune system and facilitate the effectiveness of clinical anti-cancer drugs; and the awakening of tumor dormancy to enable timely removal(Fig. 5). It is crucial to continue investigating the complex mechanisms underlying tumor dormancy and to identify new therapeutic targets to improve patient outcomes. The outstanding question is which of the three strategies to combat cancer cell dormancy is the most effective and safest treatment?

### Inducing or maintaining tumor dormancy

Modulating the tumor cell cycle to induce cell cycle organization, halt proliferation and growth, or maintain a dormant cell state may represent a novel approach for converting tumors into chronic, controllable diseases. Notably, a compound known to activate NR2F1, a regulator of tumor cell dormancy, C26, has been shown to inhibit tumor growth and metastasis by inducing tumor cell growth arrest [100]. In a recent study, NR2F1 was found to induce tumor cell quiescence by binding to the promoters of SOX9, RARβ, and p27 and inducing their expression. Specifically, upregulation of NR2F1 can activate dormant gene signals, such as SOX9 and retinoic acid receptor β (RARβ), which then activate the expression of cell cycle protein-dependent kinase (CDK) inhibitors p27 and p16. This leads to G0/G1 cell cycle arrest and cell quiescence. Interestingly, the researchers found that NR2F1 is highly enriched in ER-positive tumors compared to ER-negative tumors, and activating NR2F1 may prevent the reawakening of dormant cancer cells. Therefore, compounds that activate NR2F1 may hold promise for the treatment of tumors.

Recent studies have highlighted the close relationship between tumor dormancy and the extracellular matrix (ECM) in the tumor microenvironment. In particular, Di Martino JS et al. [39] found that dormant tumor ECM is characterized by disorganized, curled collagen fibers, while the ECM of proliferating tumors shows a straighter, more linear arrangement. When dormant tumor cells awaken, the surrounding collagen exhibits a more linear distribution. These findings underscore the critical role of the tumor microenvironment in regulating tumor dormancy and suggest that type III collagen may be particularly important in this process. Specifically, adding type III collagen to the tumor microenvironment has been shown to promote the entry and maintenance of tumor cells in a dormant state, thereby inhibiting tumor cell proliferation.

Additionally, studies have demonstrated that interferon-gamma produced by natural killer cells can help maintain tumor cells in a dormant state, an effect that has been observed in breast cancer cells [96]. Barrow AD [101] et al. have also shown that natural killer cells can inhibit tumor growth by modulating relevant cytokines. Collectively, these findings underscore the importance of both cells and the ECM in regulating tumor dormancy. Inducing or maintaining tumor dormancy represents a critical area of future research with significant implications for tumor treatment.

### Direct targeting of dormant cells

Tumor recurrence is a critical concern in cancer treatment, often resulting from the presence of dormant tumor cells that exhibit resistance to anti-proliferative chemotherapy and insensitivity to relevant signaling pathway inhibitors [42]. Dormant disseminated tumor cells (DTCs) are emerging as key targets for anti-tumor metastasis and recurrence therapy. Still, their prognosis for clinical treatment remains challenging due to drug resistance and other factors. Researchers at the Fred Hutchinson Cancer Research Center [102] have shed light on the drug resistance mechanisms of dormant DTCs, demonstrating that their resistance is unrelated to cell cycle status but rather is mediated by integrins that facilitate cancer cell adhesion to the extracellular matrix. Specifically, the researchers found that dormant DTCs express high levels of integrins and confer chemoresistance by protecting the cells from the cytotoxic effects of chemotherapy drugs. However, they also showed that inhibiting integrins could sensitize dormant tumor cells to chemotherapy. These findings highlight the potential of integrin inhibitors as a targeted therapy for tumors and suggest that further research in this area may yield promising results for improving cancer treatment outcomes. It is worth mentioning that the autophagy and senescence mechanism, as a regulatory mechanism of tumor dormancy, may be a new therapeutic approach to use it as a new entry point for tumor dormancy treatment. Currently, autophagy inhibitors are used clinically to circumvent the protective autophagy of cells and sensitize tumor cells to drugs such as radiotherapy and chemotherapy [103, 104], thus improving the anti-cancer effects of drugs. Senescence due to exposure to anticancer therapies and radiation is called therapy-induced senescence (TIS), TIS is closely related to cancer recurrence, and it has a potential role in tumor dormancy [105]. The use of drugs with anti-aging properties [106] (e.g., senolytics and senostatics) is currently being used clinically as a therapeutic strategy to eliminate persistent residual survival of tumor cells, thereby reducing the likelihood of cancer recurrence.

### Awakening tumor dormancy

The proliferation of tumor cells characterizes tumor dormancy into a quiescent state, in which the cell cycle is arrested, and they undergo a transformation into a form of immune evasion [107]. Such dormant cells exist latently in the body, increasing the likelihood of subsequent tumor recurrence or metastasis. The inactivity of these cells renders many chemotherapeutic agents, which target the S phase of the [108] cell cycle, ineffective [109]. Furthermore, the immune system fails to recognize and eliminate these cells, limiting existing therapies’ effectiveness [110]. Consequently, the reactivation of dormant cells to facilitate their recognition by the immune system, followed by the administration of clinically targeted anti-cancer drugs, may be a potential approach for treating tumors.

Various studies have demonstrated that an “inflammatory environment” acts as an “incubator” for tumor development, and chronic inflammation can lead to the awakening of dormant cells. Denk D [111] et al. have reported that inflammatory chemokines within the tumor microenvironment can stimulate dormant cancer cells’ reactivation. Neutrophils, under the influence of inflammation, have been shown to awaken adjacent dormant cancer cells in a specific manner, as observed in breast, lung, and prostate cancers [72, 112, 113]. However, the application scenarios for this approach need to be carefully evaluated. Following the reactivation of dormant tumor cells, targeted therapeutic strategies based on tumor growth status and changes in the tumor immune microenvironment should be implemented. Potential adverse consequences such as tumor metastasis and accelerated progression after tumor reactivation must be closely monitored, and appropriate countermeasures must be taken.

## Outlook

The current methods for treating tumors include surgery, radiotherapy, chemotherapy, and immunotherapy. However, these treatments often produce severe toxic side effects and are ineffective in treating metastatic tumors. As science, technology, and economic development progress, there is a growing demand for “precision medicine.” The study of tumor dormancy mechanisms has become a prominent area of research in anti-tumor research in recent years, which could be a critical requirement for achieving “precision medicine”. This area of research can provide new insights into “precision medicine.”

However, the current understanding of tumor dormancy is incomplete because there are various states of tumor dormancy, and different mechanisms lead to the same final “dormant” condition, making it challenging to define the underlying cause of dormancy. Therefore, it is crucial to continue investigating the different mechanisms of tumor dormancy to develop more effective and precise treatments for cancer. Additionally, the investigation of tumor dormancy mechanisms may provide a basis for the development of novel therapeutic strategies that target tumor cells’ dormant state.

Several therapeutic strategies have been developed to target tumor dormancy. However, the number of tumor dormancy-specific marker genes identified so far is limited, which makes it challenging to accurately determine the dormancy status of cells using existing dormancy-related genes. The availability of more tumor dormancy-specific genes could facilitate the labeling of dormant cells and tracking of their cell fate. Furthermore, tumor dormancy-specific genes are essential for evaluating the role of dormant cells in various therapies. Therefore, identifying new tumor dormancy-specific marker genes is currently a high priority in this field of research.

In conclusion, enhancing our comprehension of the fundamental characteristics and molecular regulatory mechanisms underlying tumor dormancy, coupled with the development of novel drugs and therapeutics that can interfere with dormancy induction, eliminate dormant cells, or prevent dormant cells from reactivation, could potentially serve as innovative and effective strategies for future tumor therapy.

## Data Availability

No data were involved.

## Abbreviations

ACR: Autophagy cargo receptor
ADR: Adriamycin
AHR: Aromatic hydrocarbon receptor
Akt: Protein kinase B
APC/C: Anaphase-promoting complex/cyclosome
ARHI: Aplasia Ras homolog member
CDKs: Cyclin-dependent kinase
DTC: Disseminated tumor cell
ECM: Extracellular matrix
EMMPRIN: Extracellular metalloproteinase-inducible factor
ERK: Extracellular signal-regulated kinase
Grb 2: Growth factor receptor binding protein 2
IDO: Indoleamine 2,3-dioxygenase
IFN-γ: Interferon-gamma
IFN-β: Interferon-beta
IFNAR: IFN-β receptor
IRF7: Interferon regulatory factor 7
Kyn: Kynurenine
MAPK: Mitogen-activated protein kinase
MEK: Methyl ethyl ketone
MSCs: Mesenchymal stromal cells
MLCK: Myosin light chain kinase
MMC: Mouse mammary carcinoma
mTOR: Mechanistic target of rapamycin
mTORC2: Mammalian target of rapamycin complex 2
NETs: Neutrophil extracellular traps
NR2F1: Nuclear receptor subfamily 2 group F member 1
PDK1: 3-Phosphoinositide-dependent protein kinase 1
PFKFB3: 6-Phosphofructo-2-kinase/fructose-2,6-bisphosphatase
p-FAK: Phosphorylated focal adhesion kinase
PI3K: Phosphoinositide 3-kinase
PIP2: Phosphatidylinositol 4,5-bisphosphate
PIP3: Phosphatidylinositol 3,4,5-trisphosphate
PMN: Pre-metastatic ecological niche
p-Src: Phosphorylated Src kinase
p53: Tumor protein 53(TP53)
RARβ: Retinoic acid receptor beta
SOS: Son of sevenless
SOX9: SRY (sex determining region Y)-box 9
S100: A family of calcium-binding proteins, play crucial roles in various cellular processes such as cell cycle progression, differentiation, and apoptosis.
TGF-β: Transforming growth factor-beta
TME: Tumor microenvironment
TNBC: Triple-negative breast cancer
TNF-α: Tumor necrosis factor-alpha
uPAR: Urokinase plasminogen activator receptor
YAP: Yes-associated protein
5-Fu: Pentafluorouracil
